# Accelerating Discovery for Complex Neurological and Behavioral Disorders Through Systems Genetics and Integrative Genomics in the Laboratory Mouse

**DOI:** 10.1007/s13311-012-0111-3

**Published:** 2012-03-16

**Authors:** Jason A. Bubier, Elissa J. Chesler

**Affiliations:** The Jackson Laboratory, Bar Harbor, Maine 04609 USA

**Keywords:** Systems genetics, Recombinant inbred mice, genomics, Data integration, Bioinformatics

## Abstract

Recent advances in systems genetics and integrative functional genomics have greatly improved the study of complex neurological and behavioral traits. The methods developed for the integrated characterization of new, high-resolution mouse genetic reference populations and systems genetics enable behavioral geneticists an unprecedented opportunity to address questions of the molecular basis of neurological and psychiatric disorders and their comorbidities. Integrative genomics augment these strategies by enabling rapid informatics-assisted candidate gene prioritization, cross-species translation, and mechanistic comparison across related disorders from a wealth of existing data in mouse and other model organisms. Ultimately, through these complementary approaches, finding the mechanisms and sources of genetic variation underlying complex neurobehavioral disease related traits is becoming tractable. Furthermore, these methods enable categorization of neurobehavioral disorders through their underlying biological basis. Together, these model organism-based approaches can lead to a refinement of diagnostic categories and targeted treatment of neurological and psychiatric disease.

**Electronic supplementary material:**

The online version of this article (doi:10.1007/s13311-012-0111-3) contains supplementary material, which is available to authorized users.

## Introduction

Among the critical challenges in the discovery of pharmacotherapy for behavioral and neurological disorders are the heterogeneity and comorbidity of the disorders and the diversity of mechanisms by which they arise. Shared biological mechanisms may underlie frequently comorbid behavioral disorders, and diverse etiological mechanisms may each result in the same behavioral disorder. Furthermore, environmental factors influence the structure and function of the nervous system, playing a major causal role in behavioral disorders in the same manner as endogenous genetic variation. Genetic variation leading to individual differences in neural function may also influence environmental preferences or niche selection, thus correlating particular genetic backgrounds with particular environmental exposures.

These issues have challenged those engaged in efforts to define and classify psychiatric conditions for basic research, diagnostics, and therapeutics. The definitions of these disorders in the International Classification of Diseases, Diagnostic and Statistical Manual, and other classification schemes are heavily reliant on sociocultural, subjective, and external clinical manifestations of the disorders. Therefore, such schemes may result in a poor mapping of diagnostic categories onto biological mechanisms of disease. This challenge is further compounded in the use of animal models to study disease, for which one strives for true construct validity, but relies instead on tests that were often historically devised for pragmatic factors, including pharmacological response validity and face validity. Furthermore, the validity and reliability of these assays is made difficult due to the challenges of generalizing behavioral results across testing paradigms and laboratory environments.

Patterns of comorbidity in behavioral health are considerable, and the heterogeneity of individual characteristics and diagnostic categories present challenges to precise, accurate diagnosis and alignment to effective treatment. Results from the National Epidemiologic Survey on Alcohol and Drug Related Conditions (NESARC) and other studies reveal extensive comorbidity. For example there is greatly increased prevalence of psychiatric and behavioral disorders among individuals with substance use disorders [1–10] and a corollary high prevalence of drug abuse and dependence with mental disorders [11]. Understanding the relations among diverse neurobehavioral disorders is critical to identifying the biological basis of comorbidity, developing a biologically driven classification of behavioral disorders, and identifying the precise biomolecular networks underlying comorbidity.

It is essential to be able to categorize disease, define subtypes, and operationally define robust, reliable, and valid research models to develop efficacious interventions. This may be particularly true for pharmacotherapeutics, but these issues also apply to the development and application of biopsychosocial therapies that are tailored to the specific subtypes and biological mechanisms of psychiatric disorders. As we move toward personalized and predictive medicine for neurological and psychiatric disorders, including pain, mental health, and disorders of addiction, it becomes ever more critical to accurately define and characterize particular classes of behavioral disorder. One approach to this challenge is to define disorders by, and simultaneously associate them with, underlying biological mechanisms and manifestations of the disease.

Integrative genetics and genomics are emerging strategies to implement this approach. These methods have advanced largely through mouse genetics and systems biology. They have the potential to identify and evaluate heretofore poorly characterized therapeutic targets and simultaneously associate these biomolecules to particular facets of behavioral disorders. Integrative or systems genetics applies systems biological methods including high-throughput molecular assays and network modeling to the study of population genetic variation. Studies of this type often use a single population as a reference to integrate data across a variety of biological functions and across biological scales. Recent advances in mouse genetic reference populations capture unprecedented allelic diversity and will greatly improve the power and precision of these studies [12–14]. The genetic variation inherent in the populations drives multiple traits simultaneously, enabling discovery of the common genetic basis and correlated molecular functions for a wealth of pleiotropic sequelae of genetic variation. Integrative genomics uses genes and other biomolecules as a reference with the goal of examining the shared and unique basis of disorders annotated to those biomolecules across species and experimental systems. New web-based resources, including our own GeneWeaver.org, enable the integration of genomic data across large numbers of studies and a range of model organisms [15, 16]. The global objectives of these complementary approaches are to identify the molecular underpinnings of related behavioral phenotypes, to exploit this information to define categories of related or distinct behavioral traits and to enable reclassification of behavioral disorders, based on associated molecular networks. Together, integrative genetics and genomics enable a meaningful shift from face validity to molecularly-based construct validity in the development of classification schemes, cross-species translation of disease models, and identification of specific therapeutic targets for specific manifestations of psychopathology.

## Systems and Integrative Genetics

### Overview

Integrative genetics relies on the phenomenon of gene pleiotropy. A polymorphism will cause biologically related disorders to co-vary (i.e., to be comorbid), based on a shared role for the affected gene in the underlying biological processes. The corollary to this is that distinct diseases are largely driven by distinct polymorphisms, even when they have the same behavioral manifestations. Despite convergent behavioral manifestations, such as the tendency to consume excessive amounts of alcohol among individuals with diverse underlying psychopathology, these disorders should be considered distinct phenomena when searching for biological mechanisms and therapeutic interventions. Indeed, the challenge of identifying genes for behavioral disorders has been largely one of refining phenotypic definition and genetic population composition to better associate behavioral variation with genetic diversity. Without such refinement, genetic factors account for only limited amounts of population phenotypic diversity. Integrative or systems genetics is a method used for the genetic correlation of disease-related phenotypes across individuals in order to assess their cohesion in functional categories, and for the correlation of disease-related phenotypes to underlying biological mechanisms of disease.

### Quantitative Trait Locus Analysis of Behavior in the Laboratory Mouse

The laboratory mouse has a long history in behavioral neuroscience, and the use of the laboratory mouse for the genetics of complex traits, including behavior is well-established. A variety of resources exist for performing experimental crosses of two or more strains to randomly segregate genotypes among the resulting progeny. By correlating genotypes with phenotypes in quantitative trait locus (QTL) analysis, a large number of polymorphic regions harboring trait relevant allelic variation have been defined for a wide range of behavioral phenotypes [17]. At present, there are 549 QTLs for behavioral phenotypes in the Mouse Genome Informatics database, which are largely derived from crosses of 2 inbred strains of mice [18]. A major benefit of QTL analysis is that any polymorphic feature can be implicated as the cause of variation in a complex trait, as opposed to reverse genetics methods, which involve targeted perturbation of known genes and gene products. However, there has been a critical challenge with QTL analysis. Historically, the resulting genetic loci have been large, sometimes containing several hundred candidate genes. With the discovery of many new noncoding DNA features, cryptic splice sites and other noncoding variation, the search for the cause of trait variation within these loci is even more challenging than it once appeared. Moreover, each population used in conventional mapping crosses is independently bred and it is typically impossible to retrieve a sample of mice with the same genetic configuration. Furthermore, QTL mapping does not lend itself readily to the sharing of information across experiments. In earlier studies, each panel of mice was subject to a limited number of phenotypic measures, often of highly related behavioral traits. Although the independence of mapping crosses allows independent replication of mapping results, data integration across studies is only possible through the mapped loci themselves.

### Multi-dimensionality in Mouse Genetic Reference Populations

**Fig. 1:**
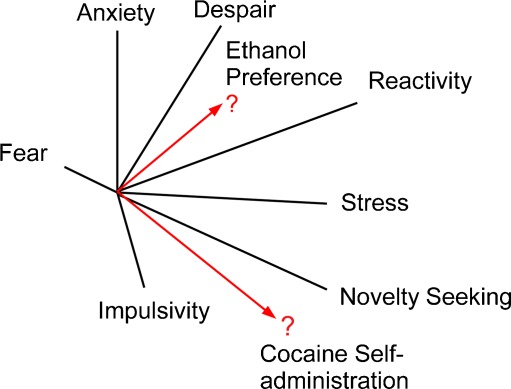
A schematic representation of the integrative multi-dimensional analysis of behavior and the relation of substance-use phenotypes to previously established factors of behavior in a reference population

Genetic reference populations (panels of recombinant inbred strains) feature the same random segregation of genetic loci found in an experimental cross. However, the population is inbred, enabling indefinite retrieval of the population for further characterization, leading to multiplicative aggregation of phenotypic data. This important characteristic allows broad multi-dimensional profiling of the population through independent studies, which also allows discovery of underlying factors of behavioral variation and comorbid disorders (Fig. 1). The integrative value of recombinant inbred strains for behavioral genetic analysis has been long appreciated [19]. Advances in computation, bioinformatics, and the proliferation of Internet-based biological resources enabled the development of the integrative Gene Network (www.genenetwork.org) system [20] for the aggregation and analysis of molecular and trait data across the recombinant inbred lines, including the largest existing set, the C57BL/6 × DBA/2 recombinant inbred (BXD RI) lines.

**Fig. 2:**
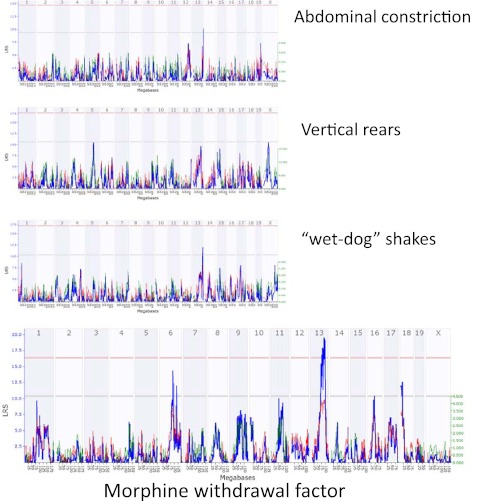
Multi-dimensional genetic analysis in the BXD recombinant inbred (RI) genetic reference population. QTL mapping of a factor predictive of naloxone-induced morphine withdrawal, naloxone (30 mg/kg i.p.) after morphine (50 mg/kg dose i.p.). Blue traces are likelihood ratio statistics. The red/green trace plots the additive effect with green representing increase or effects of DBA/2J alleles and red representing positive effects of C57BL/6J alleles. Horizontal lines represent the *p* < 0.05 (red) and *p* < 0.63 (blue) empirical significance thresholds. Analysis of 3 distinct measures of morphine withdrawal reveals no significant QTL, although suggestive loci are present. The combined analysis of multiple behaviors through a reference population reveals a significant QTL for a derived factor of these and other correlated behaviors [22]. The “Morphine Withdrawal Factor” was derived by performing a maximum likelihood factor analysis on measures from our high throughput behavioral study in the BXD RI lines

In the expanded BXD RI mouse population [21], we have recently made more than 250 measures from approximately 40 behavioral tests, including multiple traits relevant to drug and alcohol sensitivity and withdrawal, basal behavioral variation, and neurobehavioral measures reflective of stress, anxiety, despair, activity, pain sensitivity, and startle [22]. These data were all contributed to the database of phenotypes on GeneNetwork.org [23]. Using the GeneNetwork embedded QTL mapping software, quantitative trait loci that regulate each trait were detected [24]. The entire trait co-expression matrix was then subject to a factor analysis that allowed the identification of behavioral factors, which could then be correlated with other characteristics of the mouse population. For example, we used this analysis to identify a factor related to the reactive response to both auditory and thermal stimuli, and found that this factor is correlated with preference for alcohol self administration. Recent human studies applied a conceptually similar approach to identify related personality correlates of alcohol drinking [25]. Another factor appears to be highly related to diverse measures of morphine withdrawal. Although no single measures of morphine withdrawal could be mapped to a significant locus, the combination of the correlated traits improved the ability to detect a common genetic signal (Fig. 2). The identification of genes underlying these common factors of human behavior is a lengthy and expensive endeavor. Mouse genetic reference populations can be a deep and efficient resource for the discovery of the biological basis of these relations. New genetic approaches in model organisms can accelerate the discovery of the causative loci and candidate mechanisms of these correlated phenotypes, and translational bioinformatics strategies can be applied to assess the biological construct validity of the mouse model phenotypes used to identify these factors.

### Correlation across Biological Scale: Identification of Co-Expressed Traits and Genes

Systems genetics enables biological mechanisms to be associated with factors of behavioral variation *en masse*. This method integrates systems biological methods of high throughput molecular characterization and mathematical modeling of networks with the methods of systems genetics analysis. The advent of whole-genome gene expression technology and other molecular profiling techniques has enabled the deep integration of behavioral phenotypes in these populations with biomolecular traits.

The earliest systems genetics studies used genetic reference populations to map genetic loci that regulate the expression of genes. These studies found massive patterns of gene co-expression, including groups of genes that are highly correlated with behavioral traits. Because these studies broadly sampled brain gene expression and previously existing behavioral data, there has been a proliferation of systems genetics work in diverse genetic reference panels and experimental crosses. Using brain gene co-expression networks, genes, and polymorphisms have been identified that are associated with anxiety-like behavior [26], diabetes, = obesity [27], and most recently, fear conditioning [28].

### Advanced Reference Populations for Integrative Genetics

Mouse experimental crosses and simple 2 progenitor recombinant inbred populations have been a major enabling technology for the discovery of biological mechanisms of neurobehavioral phenomena, but the conventional populations have had some major drawbacks. The power and precision of the existing populations are typically very low. One strategy to improve power is to decrease segregating background noise, and in the process begin moving toward a congenic mouse population. This has been done through the creation of chromosome substitution strains, in which a chromosome from 1 mouse strain is introgressed onto the background of a different strain through a marker assisted backcrossing [29]. These mice have been used to study pre-pulse inhibition, among other measures of behavior [30], but lack locational precision without additional backcross mapping [31].

Increasing the sample size in QTL mapping across populations to several hundred mice can improve precision because each individual possesses unique meiotic recombinations that reduce the QTL size. Advanced intercross populations take advantage of the added recombination introduced at each generation [32]. Another strategy used to improve QTL precision is to perform additional crosses between inbred lines that have different recombinant ancestral haplotypes in the QTL interval [33]. These existing short haplotype regions narrow the interval and number of candidate genes. Others have taken advantage of the existing short haplotypes in the common inbred strains, alone [34], and in a panel referred to as the Hybrid Mouse Diversity Panel, in combination with the recombinant inbreds [28]. New mouse genetic reference populations make use of each of these properties to improve power and precision for genetic mapping and genetic correlation.

The distance between the founders of a mouse population affects the precise number of polymorphic loci that can be detected. Typical mouse genetic populations make use of only 2 founders from the closely related common inbred strains, and therefore possess a limited number of genetically variable loci [35]. Notable exceptions to this are the heterogeneous stock populations, several of which have been used quite extensively for behavioral genetics for QTL mapping and in the derivation of selected lines [36, 37]. Motivated by advances in systems genetics, new mouse populations are being developed. The Collaborative Cross is derived from 8 inbred founders and exhibits a tremendous degree of genotypic and behavioral diversity [13, 38]. The HS-CC [39] and The Diversity Outcross (J:DO), heterogeneous stocks bred from Collaborative Cross lines, and thus derived from the same founders, segregates this diversity randomly for many generations, leading to increasingly refined genetic loci [40].

**Fig. 3:**
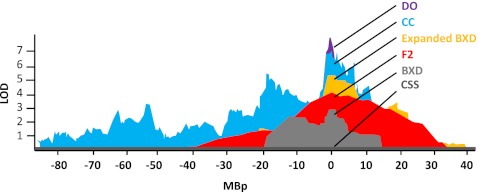
An overlay of representative QTL scans centered at their peak loci mapped with approximately 250 to 300 mice in each study. The entire chromosome containing each representative locus is shown. QTL precision is dramatically improved with new reference populations, including the Collaborative Cross, Diversity Outbred, and to a lesser extent, the expanded BXD recombinant inbred panel. A panel of consomics mice has the ability to narrow a QTL to the resolution of a single chromosome [30]. Thirty-five BXD recombinant inbred strains were used to map basolateral amygdala complex size, resulting in a significant QTL on chromosome 8 [41]. The precision of an F2 cross produces a small, broad significant QTL for bone mineral density on chromosome 7 [42]. From the expanded BXD RI population, a narrow QTL was mapped on chromosome 13 for open field rearing after cocaine [22]. Using mice from the collaborative cross breeding population a QTL for liter size was mapped to a narrow locus on chromosome 6 [14]. Early studies using the Diversity Outcross population of mice reveal high precision loci for light-dark box behavior. CC = Collaborative Cross; DO = Diversity Outbred; F2 = F2 hybrid cross; LOD = Logarithm of odds; MBp = megabase pairs

These new populations with ultra high diversity and high precision of recombination will be a tremendous advantage for behavioral and neurological studies due to the increased precision of QTL mapping (Fig. 3). It has long been speculated that the historical mouse populations, including the widely used laboratory strains, have been selected for docility, and thus constitute a narrow band of behavioral diversity. Our earliest characterization of the Collaborative Cross mouse population reveals that phenotypic diversity greatly exceeds that of the BXD RI mouse population. Furthermore, we show that by systematically intercrossing diverse laboratory mice, continuous variation in behavioral wildness can be restored, resulting in mouse models of neurobehavioral variation that more closely resemble a normal mouse population [14]. Genetic analysis in the collaborative cross reveals QTLs that are more precise, containing fewer candidate genes and polymorphisms [14].

In summary, genetic analysis in mouse populations has moved from single trait studies to broad integrative studies of multiple related phenotypes and their endophenotypes. The integration of trait data across levels of biological scale through the use of genetic reference populations enables discovery of biological co-regulation and thus, the identification of the biological basis of co-expressed traits. Those co-expressed traits may range from molecular mechanistic underpinnings of behavioral disorders to disease measures related to comorbid disorders. New mouse populations are a critical resource to boost the power and precision of these studies.

## Integrative Functional Genomics

### Overview

Integrative functional genomics provides another path to use mouse model organism data as a point of entry into biological mechanisms of pathology and comorbidity. There is a tremendous and rapidly growing amount of data coming from the widespread adoption of genomics in behavioral neuroscience and psychiatric studies. These began with early studies that mapped QTLs for behavioral traits, typically in rodent populations, but also in flies and other species. The later invention of whole genome expression profiling and expression QTL analysis have generated large sets of differentially expressed genes associated with psychiatric disorders and their model organism cognates. Expression QTL mapping studies provide yet another source of genomic data on the transcriptional effects of genetic variation in diverse processes [43]. Performing these studies in mouse genetic reference populations creates another large set of data types, resulting from gene co-expression to behavior [23]. Systematic efforts to curate experimental results and annotate genes to brain and behavioral processes represented in the Open Biomedical Ontologies [44], Gene Ontology [45], Disease Ontology [46] and Mouse Phenotype Ontology [47], the latter of which is increasingly being populated by the results of broad scale mutant and knock out phenotyping efforts. Advances in human genetics have now implicated loci across the genome with behavioral and psychiatric phenomena. Each of these behavioral and neural genomics studies are being performed in a growing array of model organisms, largely including *Mus musculus,* *Rattus norvegicus, Homo sapiens, Drosophila melanogaster, Danio rerio, Caenorhabditis elegans,* and increasingly in nonhuman primates, such as *Macaca mulatta.*

### Integrative Functional Genomics

Integrative functional genomics is an emerging data intensive approach to the matching of many genes to many behaviors and refining the results of genome scale investigations. In this method, the biomolecular entity is the reference through which data are integrated, whether it is a gene, single-nucleotide polymorphism, microRNA or other functional or nonfunctional gene product. Gene homology allows for the integration of genomic studies across species, and therefore to obtain construct valid mappings of phenomena from model organisms onto human psychological disorder. Several investigators are attempting this approach informally for small sets of genomics data to address key questions of integrative functional genomics analysis. These efforts seek to discover: 1) those genes and gene products that are consistently associated with particular disorders, 2) those that are common to multiple related disorders, 3) those that distinguish among disorders, and 4) those that are conserved across species. A less frequent application that we emphasize in our work is the development of tools to enable researchers to identify those disorders that are similar to one another through common biological substrates.

### The Wealth of Secondary Data

There is a tremendous amount of data generated from functional genomics analysis. Related to alcoholism alone, there are abstracts from more than 270 published QTL mapping studies, 9 genome-wide association studies (GWAS), and 304 gene expression publications at the time of this writing. In most genome-wide experimental paradigms results can often be distilled into a list of genes or genomic features, along with a description of the criteria describing the group of genes, such as the methods or experimental processes by which the list was generated. Although some applications integrate genomic data at the level of primary data generated from analytic equipment, many others, including our own approach, integrate experimental results that are derived in part from analysis and other interpretive decisions made by the investigator. For example, there are disparate archives for raw expression data (Gene Expression Omnibus; http://www.ncbi.nlm.nih.gov/geo/), QTL Archive mapping data (http://www.qtlarchive.org/), and inbred strain phenotypes (http://phenome.jax.org/ and http://GeneNetwork.org). Information regarding the comparison made, or the results of the study, are explicit in metadata, but only implicit in the raw data until deep analysis occurs. GeneWeaver.org stores and integrates experimental results or “secondary data,” by storing lists of genes and scores in the form of gene lists that one might derive from the previously described resources, including *p* values or q-values from differential expression experiments, a list of positional candidate genes from the confidence interval around a QTL, or a list of co-expressed genes and their correlation statistic.

### The Current State of Functional Genomics Data for Data Integration

The unfortunate challenge created by this wealth of secondary data is that it is all largely stored in a noncomputable form. Each of these studies report massive amounts of information regarding the functional roles of genes and other biomolecular entities in diverse processes; however, for most readers of the literature, it is technically challenging to summarize and integrate these findings across studies. Model organism databases store functional information, expression data, mapping data, and reference population phenotypes in highly integrated but separate repositories for each species. Domain centered databases typically store information on the role of genes and gene products in specific biological functions (e.g., Synapse DataBase, http://syndb.cbi.pku.edu.cn/ [48]; Ethanol-Related Gene Resource, http://bioinfo.mc.vanderbilt.edu/ERGR/ [49], Knowledgebase for Addiction-Related Gene [KARG], http://karg.cbi.pku.edu.cn/ [50], and PainGenesdb, http://www.jbldesign.com/jmogil/enter.html [51]). Each of these resources is valuable for its specific audience, but they may provide few analytic capabilities, interoperability, and integration with other data sources for the combination and comparison of results. Efforts to create registries of bioinformatics resources, such as these, have been helpful, and data federation enables cross database queries. Perhaps the most challenging data of all are the many publication tables and manuscript supplements that are typical of functional genomics studies. Because genomic data are stored in widely disparate manners, and methods to integrate the data require a fair amount of facility with diverse informatics tools and approaches, it remains difficult to apply these phenomenal data resources to the fundamental question of which processes share common and distinct biological substrates, and hence, which behavioral disorders should be classified together for the development of more precise diagnostics and targeted therapeutics.

### Creating an Integrative Platform

In Gene Weaver (http://geneweaver.org) [15, 16], we have created a web-based software system and data repository for broad, large-scale, integrative functional genomics analysis. The system is free to use, and with registration, it allows advanced features for long-term storage and access controlled sharing of data and results. In most cases, functional genomics results can be stored assets of biomolecules, most commonly genes, and the processes that these molecules are associated with. Gene sets are stored in the repository, retrieved by user queries of gene or terms, such as “alcoholism” or “striatum,” and analyzed using a variety of tools.

The current gene set repository in Gene Weaver contains more than 48,000 gene sets consisting of more than 80,000 genes from 7 species. A summary of the search results for alcoholism, cocaine, or other drugs of abuse or behavioral disorders identifies ~5,000 gene sets. These gene sets are curated data that has been submitted or imported from public resources, including the drug-related gene database of the Neuroscience Information Framework [52], Gene Network [53], and the Comparative Toxicogenomics Database [54]. Positional candidates of behavior-related mouse QTL have been obtained from the Mouse Genome Database [18], and gene expression in various brain regions has been obtained from the Allen Mouse Brain Atlas [55]. The Gene Weaver user community can also submit experimental results and other gene set centered data for curation into the public database.

### A Generalized Network-Based Approach

Gene Weaver uses groups of genes that have been experimentally associated with neurobehavioral phenomena as the basis of data integration. A bi-partite (two-part) network of genes and functions is constructed and explored to find the common and unique genes related to sets of behavioral processes. In this network, binary associations of genes-to-functions are indicated as edges between the two types of nodes. Each gene is mapped onto its homologs across all species, allowing a combination of experiments from several species. Although this approach may seem somewhat trivial, for large sets of genes and phenotypes the enumeration of completely connected groups of gene sets and their largest common intersection from thousands of experiments is a computationally intensive process facilitated by advanced algorithms. There are many applications of analyzing such a network, largely through the evaluation of the intersections among sets of genes for similar and distinct processes.

#### Finding Highly Ranked Genes

In the simplest application of the integrative functional genomics strategy, one merely combines the results of many independent experiments of related phenomena to find those genes that are conserved across species and frequently associated to the function in question. Cross-species analyses of pain-related phenotypes by others using smaller collections of studies have revealed highly conserved pain genes [56], although some interpret the low rates of overlap across species more negatively [57]. At the present time, it is clear that the experimental data are quite sparse and a means of connecting large numbers of diverse studies is required. Using a large bi-partite graph, we have combined 166 gene sets reflective of genome wide pain studies, and we identified genes that were found in nearly 10% of these studies, including well-studied genes, such as *Trpa1*, *Trpv1,* and *Cacna1a*, among several less well-studied targets. It is important to note that the inputs to these analyses include broad-based genome wide studies where any gene is a viable candidate, rather than those studies driven by prior gene centered knowledge.

#### Refining Genetic Loci

Aggregate functional genomics data has also been used successfully to refine QTL positional candidate loci [58]. In this application, mouse genetic loci are refined through the systematic compilation of genomic data from studies of related functions. This strategy enables refinement of large sets of candidate genetic loci to a smaller pool of highly prioritized functional candidates for which evidence supports a role in the complex trait of interest.

#### Exploring the Gene Neighborhood

Exploring the neighborhood around known genes is a powerful approach to identifying additional genes that may play a similar role in disease. For example, in a strategy similar to that used by McGary et al. [59], starting with genes known to be associated with autism in humans, one can search for all gene sets containing homologs of autism genes in the mouse. From this search, it is practical to identify those genes that are highly connected to the same sets of genes as are the autism connected genes [60].

#### Finding Related Biobehavioral Functions Through Shared Genomic Substrate

Gene set centered data can ultimately be applied to search for related biological processes based strictly on the genes to which they are connected. It is through this strategy that we expect to become able to define the biological bases of trait comorbidity by defining the shared molecular processes underlying comorbid diseases. Once such networks are identified, experimental validation of joint roles of genes in multiple comorbid diseases is practical. *Cacng1* has been identified as a gene involved in chronic pain in mice and humans [58]. In the Gene Weaver system, a user can query for this gene and identify those genes that are highly connected to similar gene sets using a “guilt-by-association” approach. *Cacng1* is found within 164 gene sets. Among those gene sets, a ranking of the most common members reveals genes that are putatively similar in function to *Cacng1* (Table 1).

**Table 1:** Gene similarity to cacnag2 based on aggregate genomic studies in diverse species

Gene symbol (*Mus* *musculus*)	Gene name	Number of shared gene sets
*Cacna1a*	Calcium channel, voltage-dependent, P/Q type, alpha 1A subunit	95
*Cacna1c*	Calcium channel, voltage-dependent, L type, alpha 1C subunit	88
*Cacna1b*	Calcium channel, voltage-dependent, N type, alpha 1B subunit	84
*Grin1*	Glutamate receptor, ionotropic, NMDA1 (zeta 1)	79
*Trpv1*	Transient receptor potential cation channel, subfamily V, member 1	77
*Cacnb4*	Calcium channel, voltage-dependent, beta 4 subunit	76
*Cacna2d2*	Calcium channel, voltage-dependent, alpha 2/delta subunit 2	73
*Kcnma1*	Potassium large conductance calcium-activated channel, subfamily M, alpha member 1	72
*Chrna7*	Cholinergic receptor, nicotinic, alpha polypeptide 7	71
*Drd2*	Dopamine receptor D2	71
*Cacng2*	Calcium channel, voltage-dependent, gamma subunit 2	68
*Scn9a*	Sodium channel, voltage-gated, type IX, alpha	68
*Cacna1d*	Calcium channel, voltage-dependent, L type, alpha 1D subunit	67
*Grin2b*	Glutamate receptor, ionotropic, NMDA2B (epsilon 2)	67
*P2rx4*	Purinergic receptor P2X, ligand-gated ion channel 4	67
*Scn8a*	Sodium channel, voltage-gated, type VIII, alpha	67
*Slc6a4*	Solute carrier family 6 (neurotransmitter transporter, serotonin), member 4	67

### Growing Beyond Existing Knowledge from Within Functional Genomics Data Sets

The enormous potential of integrative functional genomics lies in its ability to grow beyond existing knowledge of widely studied genes. Functional genomics studies carry with them the burden of validation of large numbers of poorly supported results for genes that may have no reported associations to particular neurobehavioral phenomena. However, through aggregated experiments of diverse types, it becomes evident that some of these poorly characterized biomolecules are very frequently associated with related biological phenomena.

**Fig. 4:**
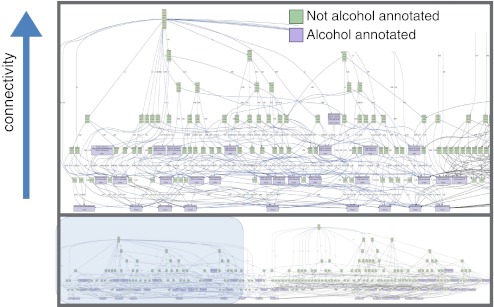
Convergent evidence for poorly studied genes associated with alcoholism. This network was constructed from 31 alcohol-related gene sets based on 3 major experimental data types: (i) QTL candidate genes, (ii) GWAS candidates, and (iii) differentially expressed genes from microarray experiments. These data came from 5 species (fruit fly, zerbrafish, mouse, monkey, and human). At the top of the tree are nodes representing multi-way intersections of the gene sets, and at the bottom are the individual genesets. The map of all gene set intersections contained more than 100 nodes. Bootstrapping was applied to reduce the complexity. Purple shading indicates nodes that contain genes previously associated with human alcoholism or mouse alcohol-related phenotypes. This analysis reveals that although many genes have been identified in specific studies of alcoholism, the most highly represented genes among the 31 data sets are not currently annotated to alcoholism

For example, we have undertaken an analysis of 31 functional genomics results from diverse studies of alcohol-related phenotypes (Fig. 4). Using our “Phenome map” tool in the Gene Weaver system, we were able to find those genes that were present in high order intersections of alcohol-related data sets, including mutant alleles annotated to alcohol-related phenotypes in laboratory mice, and human alcoholism-related genes from GWAS studies. The results are striking in that genes that have been previously associated with alcoholism occur in few gene lists derived from functional genomics experimental results, whereas genes resident in very high order intersections are as yet very poorly characterized. New technologies provide a phenomenal ability to move beyond a few well-studied targets and systems, and integrative functional genomics gives us an ability to synthesize and prioritize across numerous disparate experiments. These approaches may be extended to any area of neurobehavioral inquiry.

## Harnessing the Power of Functional Genomics in Laboratory Mice for the Identification of Novel Therapeutic Targets

For the past decade, advances in integrative systems genetics and functional genomics are complementary strategies for refining the discovery of genes associated with neurobehavioral phenomena, both of which have the potential to extract genes that are explicitly involved in comorbid disorders. The development of new, high resolution genetic reference populations, and the systems genetics analysis approaches for use of these populations, are enabling behavioral geneticists an unprecedented opportunity to address questions of the molecular basis of psychiatric disorders and their comorbidities. Integrative genomics augments these strategies by enabling the informatics-assisted rapid translation of candidate gene prioritization and functional comparison. Ultimately, through these approaches, the underlying biological basis of shared and disjoint neurological and psychological processes can be identified and applied to the refinement of diagnosis and treatment.

## Electronic supplementary material

Below is the link to the electronic supplementary material.

ESM 1 (PDF 511 kb)
