# Supplementary material for: Accelerating Discovery for Complex Neurological and Behavioral Disorders Through Systems Genetics and Integrative Genomics in the Laboratory Mouse
Source: Neurotherapeutics. 2012 Mar 16;9(2):338–48. doi: 10.1007/s13311-012-0111-3 (PMC3325414; doi:10.1007/s13311-012-0111-3)
Supplement: Supplementary file 1 — (PDF 511 kb) [file 13311_2012_111_MOESM1_ESM.pdf]

Please wait...

If this message is not eventually replaced by the proper contents of the document, your PDF viewer may not be able to display this type of document.

You can upgrade to the latest version of Adobe Reader for Windows®, Mac, or Linux® by visiting http://www.adobe.com/products/acrobat/readstep2.html.

For more assistance with Adobe Reader visit http://www.adobe.com/support/products/acrreader.html.

Windows is either a registered trademark or a trademark of Microsoft Corporation in the United States and/or other countries. Mac is a trademark of Apple Inc., registered in the United States and other countries. Linux is the registered trademark of Linus Torvalds in the U.S. and other countries.
